# Low-Impedance 3D PEDOT:PSS Ultramicroelectrodes

**DOI:** 10.3389/fnins.2020.00405

**Published:** 2020-05-19

**Authors:** Peter D. Jones, Anastasiya Moskalyuk, Clemens Barthold, Katja Gutöhrlein, Gerhard Heusel, Birgit Schröppel, Ramona Samba, Michele Giugliano

**Affiliations:** ^1^Natural and Medical Sciences Institute (NMI) at the University of Tübingen, Reutlingen, Germany; ^2^Theoretical Neurobiology & Neuroengineering, University of Antwerp, Antwerp, Belgium; ^3^joimax GmbH, Karlsruhe, Germany; ^4^Bosch Sensortec GmbH, Reutlingen, Germany; ^5^NMI Technologietransfer GmbH, Reutlingen, Germany; ^6^Neuroscience Sector, International School for Advanced Studies (SISSA), Trieste, Italy

**Keywords:** ultramicroelectrodes, PEDOT, electrodeposition, neurotechnology, MEA

## Abstract

The technology for producing microelectrode arrays (MEAs) has been developing since the 1970s and extracellular electrophysiological recordings have become well established in neuroscience, drug screening and cardiology. MEAs allow monitoring of long-term spiking activity of large ensembles of excitable cells noninvasively with high temporal resolution and mapping its spatial features. However, their inability to register subthreshold potentials, such as intrinsic membrane oscillations and synaptic potentials, has inspired a number of laboratories to search for alternatives to bypass the restrictions and/or increase the sensitivity of microelectrodes. In this study, we present the fabrication and *in vitro* experimental validation of arrays of PEDOT:PSS-coated 3D ultramicroelectrodes, with the best-reported combination of small size and low electrochemical impedance. We observed that this type of microelectrode does not alter neuronal network biological properties, improves the signal quality of extracellular recordings and exhibits higher selectivity toward single unit recordings. With fabrication processes simpler than those reported in the literature for similar electrodes, our technology is a promising tool for study of neuronal networks.

## Introduction

Recording and mapping the electrical activity of neurons *in vivo*, with adequate spatiotemporal resolution to capture both their action potentials and complex synaptic interactions, represents the holy grail of neurotechnology. Disentangling neuronal electrical activity and network communications is in fact a key step towards an in-depth understanding of the nervous system during behavior, sensation, memory, and cognition. Often, advanced technological routes are first explored and validated with reduced *in vitro* models such as brain tissue slices and dissociated cell cultures. Refinement and technology optimization *in vitro* is thus pivotal for later translating the prototypes to an *in vivo* nervous system, and two main electrophysiological approaches were first devised *in vitro*: (1) intracellular recordings from single neurons, characterized by a high temporal accuracy, selectivity and sensitivity but low spatial resolution while causing irreversible cell damage; and (2) extracellular recordings by microelectrode arrays (MEAs), capable of simultaneously accessing the electrical activity from a large number of cells over long time periods, but lacking sensitivity to subthreshold membrane potentials. Achieving the sensitivity and selectivity of intracellular and patch-clamp techniques from a large number of cells by non-invasive means is thus an intense field of research, although definitive technological solutions have yet to be discovered despite promising recent progress (Spira et al., 2018). Further scientific studies, focused on electrical, electrochemical, physical, and physicochemical properties of microelectrodes and cells are therefore urgently needed.

From an electrical perspective, an ideal recording microelectrode would be a zero-area point to increase spatial selectivity while avoiding spatial averaging, with a negligible electrical impedance to minimize thermal noise (Humphrey and Schmidt, 1990; Wellman et al., 2018). An intrinsic benefit of small electrodes is their improved signal fidelity due to reduced spatial averaging (Buitenweg et al., 2002; Viswam et al., 2019). In the context of resolving the activity of single cells, “zero-area” should be subcellular (i.e. much smaller than 10 μm), while nowadays most commercial MEAs have electrode diameters above 10 μm and thus do not meet this strict definition. This applies to sparse microelectrode arrays, while smaller electrodes additionally enable increasing the spatial resolution in dense arrays (e.g. CMOS MEAs). However, smaller electrodes require additional care to ensure low electrochemical impedance and thermal noise, and have stricter requirements for shunt capacitance of electrical connections (Robinson, 1968).

Electrodes resolving single-cell activity often measure extracellular action potentials of several nearby cells (e.g., within a range of 150 μm) (Marblestone et al., 2013), so that individual detected signal sources must be disentangled by data analysis and post-processing. Spike sorting is one of such methods and it improves our understanding of network activity by isolating the spike trains of putative single cells, although requiring higher sampling rates (e.g., 25 kHz) to resolve spike shapes, thus increasing the amount of data to be stored and analyzed. In this respect, real-time data interpretation, required in some “closed-loop” applications (e.g., neuroprosthetics), is more challenging and long recording sessions encounter practical limitations in terms of data storage (Navajas et al., 2014). Recent results with small-sized microelectrodes have demonstrated how an improved electrical coupling of cell membranes to the microelectrodes could significantly decrease the distance between the membrane and the electrode surface and thus increase the effective sealing resistance at the interface, improving the selectivity and in principle removing the need for spike sorting (Ojovan et al., 2015).

Even when single cells are isolated, extracellular recordings can hardly discriminate between action potentials of excitatory and inhibitory neurons. Statistical metric-based approaches to distinguish excitatory from inhibitory neurons had been already proposed (Becchetti et al., 2012), but combinations of spike waveforms and timing parameters from extracellular recordings do not easily allow distinguishing GABAergic interneurons from non-GABAergic neurons *in vitro* from their electrophysiological signature alone (Weir et al., 2014). Recently, a CMOS-based array of 4096 nanoelectrodes was reported to be capable of identifying excitatory and inhibitory synaptic connections by means of intracellular recordings, achieved by electroporation of neuronal membranes (Abbott et al., 2019).

In fact, it is today well accepted that microelectrodes are not passive “observers”. Their shape and surface chemical properties can enable recognition by cells and alter the electrical coupling between the cell membrane and the electrode. Nonetheless, an ideal and robust strategy to fully access intracellular electrical potentials by extracellular microelectrodes has not yet been discovered. Vertical nanowires (Almquist and Melosh, 2010; Robinson et al., 2012; Wesche et al., 2012; Xie et al., 2012; Angle et al., 2014; Lin et al., 2014; Qing et al., 2014; Dipalo et al., 2017) or microscale mushroom-shaped electrodes (Hai et al., 2010b; Santoro et al., 2013) have most widely been used to improve cell–electrode coupling, up to the point of revealing intracellular signals (Spira et al., 2018). Nanowires are intended to discretely penetrate the cell membrane, while “mushrooms” may mimic the shape of dendritic spines and encourage close contact by natural membrane-engulfment processes. Both methods were shown to achieve attenuated intracellular-like recordings, demonstrating signal-to-noise ratios, signal amplitude, and invasiveness on a spectrum between classical extracellular and intracellular recordings (Guo, 2019). Most of the promising results in the recent literature rely on active *perforation* of cell membranes (e.g., by electrical or optical means) to temporarily expose the microelectrode to the cell’s cytosol and thus to the intracellular electrical potential, even with the risk of non-recoverable cellular damage. Biological functionalization of the microelectrode surface has also been demonstrated to be beneficial (Hai et al., 2010a), so that the active recruitment of ion channels to the junctional area of the membrane might explain the spontaneous (i.e., unstimulated) intracellular-like signals (Shmoel et al., 2016). A small proportion of intracellular-like recordings with gold mushroom-shaped microelectrodes displayed unusually large peak-to-peak amplitudes for neuronal signals, above 5 mV, which were hypothesized to result from the spontaneous relocation of passive ion channels to the part of the membrane facing the electrode, thus effectively lowering the junctional membrane impedance (Shmoel et al., 2016). Promising results of mushroom-shaped electrodes have benefited from the stability and inertness of gold microstructures and their ease of use in microfabrication, despite suffering from its high electrochemical impedance. We argue below that the spike amplitudes above 5 mV reported by Shmoel et al. (2016) could even have been attenuated by capacitive shunting phenomena and would have revealed even stronger signal amplitudes if the microelectrodes had considerably lower impedances. Fully understanding the mechanisms underlying such unusually strong recordings is a promising path to engineering reliable MEA-based intracellular recordings, ultimately enabling stable, long-term future measurements.

In this paper, we describe and experimentally validate *in vitro* the use of conductive polymer coating on 3D ultramicroelectrodes, fabricated with a near-ideal size of 2 μm. The 3D structure of our electrodes increases surface area while maintaining minimal lateral size, and their coating with poly(3,4-ethylenedioxythiophene):polystyrene sulfonate (PEDOT:PSS) reduced the electrochemical impedance, achieving acceptable thermal noise levels. We ultimately successfully achieved electrode dimensions down to 1 μm by means of optical lithography, thus demonstrating an economical fabrication route in contrast to similar results relying on electron beam lithography (Weidlich et al., 2017). In comparison to CMOS MEAs (Abbott et al., 2019), our MEAs are fabricated on transparent glass substrates, allowing the easy combination with transmitted light microscopy. While PEDOT coatings previously decreased the impedance of 15-μm-large microelectrodes (Ludwig et al., 2011), here we demonstrate for the first time how PEDOT:PSS coating of 3D ultramicroelectrodes allows a similar final result. Preliminary data from this work were presented at a conference (Jones et al., 2016). Here, we detail a robust production process for serial fabrication of *in vitro* probes, and experimentally validate their operation, stability, and suitability for neural electrophysiology.

## Materials and Methods

### Fabrication

**FIGURE 1:**
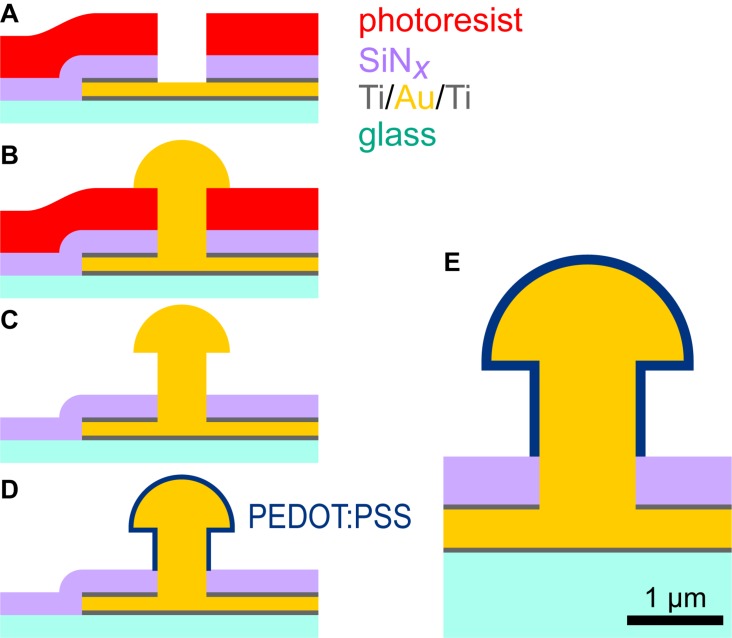
Fabrication of PEDOT:PSS ultramicroelectrodes. Conducting traces (300 nm of Ti/Au/Ti) and a SiN*_x_* insulator (400 nm thick) were patterned on glass substrates **(A)**. The photoresist mask (∼2 μm) for etching of the insulator was additionally used as the electroplating template, although etching reduced its height **(B)**. Removal of photoresist produced a 3D gold ultramicroelectrode **(C)**. Electrodeposition of PEDOT:PSS produced a conformal coating approximately 100 nm thick **(D)**. Our targeted shape was 3D with a stalk diameter of 1 μm and head diameter of 2 μm **(E)**. This drawing shows ideal geometries and illustrative dimensions.

Fabrication of microelectrode arrays (MEAs) for *in vitro* applications, featuring PEDOT:PSS-coated ultramicroelectrodes, was achieved by adapting the technological processes employed for standard MEA fabrication. The key steps are illustrated in Figure 1.

Briefly, MEAs with a large conventional internal reference electrode and 59 ultramicroelectrodes, arranged in an 8 × 8 square grid with a pitch of 200 μm, were fabricated on 1-mm-thick, 49 × 49 mm^2^ borosilicate float glass substrates. Conducting traces were produced by sputtering 300 nm of titanium and gold (Z550, Leybold, Cologne, Germany), defining an S1818 etch mask by photolithography (Gamma, SÜSS MicroTec SE, Garching, Germany), and plasma etching (CF_4_ and Ar, Z550). Silicon nitride (SiN_x_, ∼400 nm) was deposited by PECVD (Plasmalab 800, Oxford Instruments, Abingdon, United Kingdom) for insulation. The insulator and upper Ti layer were etched with CF_4_ and Ar plasma (Plasmalab 800) with a ∼2 μm AZ ECI 3027 resist mask (developed in diluted AZ 351B), which further served as a template for electrodeposition (with reduced height due to non-ideal etch selectivity). Achieving 1 μm feature sizes required advanced optimization of the lithography parameters. Photomasks with 0.75 μm features were used to obtain diameters down to 1 μm.

Before electroplating, microelectrodes were electrochemically cleaned by voltage cycling in sulfuric acid (0.5 M H_2_SO_4_, 0.4–1.4 V vs. Ag/AgCl, scan rate 300 mV/s). In this step, cyclic voltammograms were measured for individual electrodes to confirm whether etching had exposed gold. 3D gold ultramicroelectrodes were individually electroplated (VMP3 multichannel potentiostat, Bio-Logic SAS, Seyssinet-Pariset, France) from a gold electrolyte (NB Semiplate Au 100, NB Technologies GmbH, Bremen, Germany) heated to 40°C. A current of –0.4 nA was applied and the plating time was adapted according to the desired electrode size. Typical dimensions of 1 μm stalk height, 1 μm stalk diameter, and 1.5–2.0 μm cap diameter required ∼85 s. After electrodeposition, photoresist was removed by stirring substrates in 0.5 M KOH at 50°C for 5 min.

The gold ultramicroelectrodes were coated with PEDOT:PSS via electrodeposition (Gerwig et al., 2012). Prior to PEDOT:PSS coating, MEAs were treated with a 100 W air plasma for 2 min (Harrick plasma cleaner, Ithaca, NY, United States) and microelectrodes were again cleaned by voltage cycling in sulfuric acid; here, the voltage was applied simultaneously to all electrodes. The electrodes were rinsed then immersed in an aqueous solution of 1% PSS with 0.03 M 3,4-ethylenedioxythiophene (EDOT). A constant current of 0.2 nA (∼0.02 nA/μm^2^) was applied for various time intervals, lasting up to 30 s, to deposit charges of up to 6 nC.

### Electrochemical Characterization

Measurements were made with a Pt mesh counter electrode in phosphate-buffered saline (PBS). Impedance spectra were acquired from 1 Hz to 100 kHz with an amplitude of 10 mV. The electrode thermal noise was measured with a ME2100 amplifier (Multi Channel Systems MCS GmbH, Germany).

### Optical and Scanning Electron Microscopy

Optical images were acquired with an upright Olympus microscope and a 100x objective lens. Focused-ion beam (FIB) milling and scanning electron microscopy (SEM) were performed with a Zeiss AURIGA CrossBeam. Samples were sputtered with a thin layer of AuPd for conductivity prior to SEM. FIB cross-sections revealed precise 3D and internal structures.

### Stability

MEAs were stressed while their stability was monitored by optical microscopy and impedance measurements. Stresses included sonication in an ultrasound bath for 30 s, treatment with air plasma for 30 s, incubation in 96% ethanol for 14 h and submersion in PBS at 37°C.

## Cell Cultures, Electrophysiology, and Data Analysis

### Dissociated Cell Cultures

The experiments were carried out in compliance with the European Community Council directive and approved by the Animal Ethics Committee of the University of Antwerp. Prior to cell seeding, all MEAs were treated with air plasma (∼10 W for 90 s) in a plasma cleaner (Zepto BRS, Diener electronic GmbH, Ebhausen, Germany), sterilized with 70% ethanol for 30 min, rinsed in Milli-Q water for 30 min, covered with polyethyleneimine at room temperature overnight (PEI, 0.1% wt/vol in Milli-Q water, Sigma-Aldrich, Germany), then rinsed with Milli-Q water and air-dried for 30 min. Neuronal cultures were prepared from cortical or hippocampal neurons from newborn rats using standard procedures. Briefly, the skull was removed, and the brain isolated. The brain area was dissected out and cut into small pieces which were incubated for 15 min at 37°C in 0.025% trypsin (Sigma-Aldrich, Germany) solution. After enzymatic treatment the tissues were washed and mechanically dissociated. The cell suspension was diluted in order to control final density of seeding and were plated on MEAs with initial density of 6500 cells/mm^2^. Cells were maintained in minimum essential medium (MEM), containing 10% horse serum, and cultured in a 5% CO_2_ incubator at 37°C and 95% humidity. At DIV3 cells were incubated with 1 μM arabinosylcytosine for 24 h. Every 2 days half of the culture medium was replaced with fresh, pre-warmed medium and, starting from DIV12 onwards, the replacing of culture medium was performed with serum free medium.

### Extracellular Electrophysiology and Spike Sorting

Arrays of PEDOT-coated ultramicroelectrodes were tested in comparison to standard commercial MEAs (60MEA200/30iR-ITO-gr, Multi Channel Systems MCS GmbH, Reutlingen, Germany) containing 60 titanium nitride (TiN) planar microelectrodes, each with a diameter of 30 μm and with the same 8 × 8 layout and 200 μm pitch. We carried out recordings of the spontaneous neuronal activity at 37°C and 5% CO_2_ for at least 30 min, starting from 7 and continuing up to 35 days *in vitro*. For each electrode the extracellular voltage was detected and amplified by a MEA-1060-Up-BC multichannel amplifier, with a 1–3000 Hz bandwidth and an amplification factor of 1200 (MCS GmbH). Raw analog signals were acquired with a sampling frequency of 25 kHz and converted to digital signals at a resolution of 16 bits, using an A/D converter (MCCard, MCS GmbH), leading to an optimal representation of the spike waveform. Slow components and high frequency noise of the digital signals were filtered out by a band-pass filter, between 300 and 3000 Hz. Filtered signals were stored on a disk for offline data processing. Action potential detection was performed with QSpike Tools (Mahmud et al., 2014) using for each channel an individual threshold, automatically calculated as five times the standard deviation of the “background” noise (Quiroga et al., 2004). Each recorded channel with a detected neuronal spiking activity higher than 0.5 Hz was considered as an “*active”* electrode. All spike waveforms were aligned to the peak and represented by 64 data points (i.e., 2.56 ms). Spike times and spike waveforms were stored on disk for subsequent analyses, performed by custom scripts in MATLAB (The MathWorks, Natick, MA, United States). Dimensionality reduction was done with wavelet transforms by extracting distinctive features from the detected spike waveforms (Quiroga et al., 2004). This time-frequency decomposition of the signals was performed by using Haar wavelets. The selection of the best separating wavelet coefficients was done automatically, using a Kolmogorov-Smirnov test for normality, and the less normally distributed wavelet coefficients were chosen. For spike sorting we used the superparamagnetic clustering procedure (Quiroga et al., 2004). Briefly, this unsupervised method allows to group the chosen wavelet coefficients into clusters, using only one parameter, called “clustering temperature.” In case of “low” temperature, all data is grouped into one cluster, while increasing temperature leads to split of the dataset into many clusters. An optimal middle range or “superparamagnetic” temperature exists, when the data is assigned to few large clusters. After this step, spikes with the different shapes were grouped into subsets, corresponding to the isolated neuronal units.

### Statistics

Normality of the distribution was verified by the Lilliefors test. For normal distributions data are shown as the mean ± standard error of the mean (SEM); statistical significance between groups is assessed using a two-way ANOVA and post-hoc Fisher’s procedure. Values *p* < 0.05 (^∗^) and *p* < 0.01 (^∗∗^) were considered as significant differences.

## Results

### Fabrication

We successfully fabricated arrays of 59 PEDOT:PSS-coated, mushroom-shaped gold ultramicroelectrodes. Small sizes of ultramicroelectrodes are known to be critical for a tight contact with cells’ membranes (Ojovan et al., 2015). By photolithography and electroplating, our ultramicroelectrode stems had diameters as small as 1 μm. These dimensions approach the smallest resolvable feature size for contact lithography, which is on the order of $W_{\min}\approx \sqrt{\lambda d}$ for wavelength λ and mask–substrate distance *d* (Plummer et al., 2000). We predicted *W*_*min*_≈ 950 nm with a resist thickness of 2 μm and unfiltered emission from a Hg lamp of 300 to 450 nm. Approaching this limit required photomask feature sizes of 750 nm, and optimization of spin-coating, exposure, and development processes. Etching of the insulator was optimized to reveal the gold traces while avoiding overetching that would enlarge ultramicroelectrode diameters.

Cyclic voltammograms in H_2_SO_4_ not only cleaned the gold ultramicroelectrodes but also allowed us to identify if plasma etching had successfully exposed the gold. The peak at ∼0.6 V *versus* Ag/AgCl due to reduction of chemisorbed oxygen reflects the gold surface area, with charge for polycrystalline gold of 3.9 pC/μm^2^ (Trasatti and Petrii, 1992). The area of gold exposed prior to gold electroplating was <1 μm^2^. Residual Ti at the perimeter of the gold area affected the specific area (as revealed by FIB cross-sections).

The caps of each ultramicroelectrode could be precisely controlled in size by current-controlled electroplating. Electrodeposition parameters and temperature critically affected the structure of the electrodes. For example, gold deposition with -1.5 nA produced ultramicroelectrodes with voids in their stems, as revealed by FIB cross-sections. In many cases, such ultramicroelectrodes remained stable through fabrication, even with these invisible voids. Nonetheless, ensuring void-free electrodes was important to us in order to improve the device stability for biological applications. Best results without voids were obtained when using currents of −0.4 nA.

Conformal PEDOT:PSS coatings were produced by adapting established methods (Gerwig et al., 2012). After attempting electrodeposition of composites of PEDOT:PSS with carbon nanotubes (CNT) as reported by Gerwig et al. (2012) we observed uncontrolled formation of PEDOT:PSS/CNT structures extending tens of micrometers away from the gold electrodes. We assumed that this effect was caused by adsorption of CNTs on the SiN*_x_* insulator. We therefore used PEDOT:PSS without CNTs.

### Electrochemical Characterization

**FIGURE 2:**
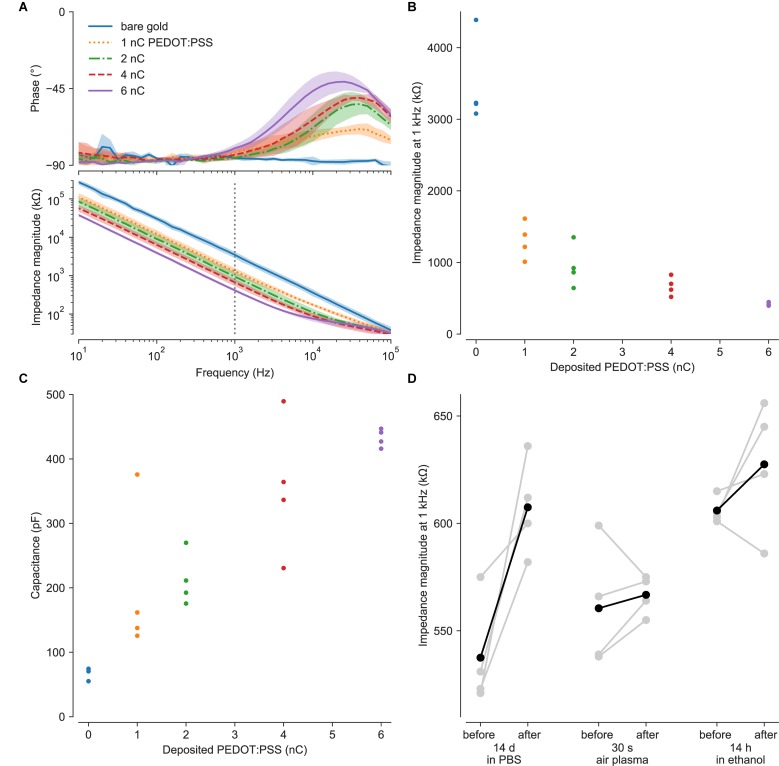
Electrochemical characterization. **(A)** Coating with PEDOT:PSS decreased the electrochemical impedance. Measurements of impedance of bare gold ultramicroelectrodes were compromised by the shunt capacitance of the insulator. **(B)** Increasing PEDOT:PSS deposition reduced impedance at the key frequency of 1 kHz. **(C)** Equivalent capacitances were fitted over the frequency range most relevant for extracellular electrophysiology (1–5000 Hz), where the electrodes had predominantly capacitive behavior with phase near −90°. **(D)** PEDOT:PSS coatings survived soaking in PBS at 37°C, exposure to air plasma, and ethanol disinfection. Degradation or removal of PEDOT:PSS would have reverted the electrodes to the value of several MΩ impedances (at 1 kHz) of the uncoated electrodes. Points in B and C indicate values from individual electrodes. Gray points in D indicate paired measurements for single electrodes; black points are averages as a visual guide.

Impedance measurements revealed as expected low impedances of the PEDOT:PSS-coated ultramicroelectrodes, in consideration of their size (Ludwig et al., 2011; Gerwig et al., 2012). Impedances decreased with increasing PEDOT:PSS deposition (Figures 2A,B), and were 442 ± 86 kΩ at 1 kHz (*n* = 105) with 6 nC of PEDOT:PSS. Consistent with these values, the median electrode noise was 6 μV_*rms*_. Approximately 90% of electrodes were functional (here, 105/118 from two typical MEAs), based on the impedance magnitude below 1 MΩ at 1 kHz. Process optimization would be needed to reach 100% yield expected for commercial-grade MEAs.

For ultramicro- or nanoelectrodes, spreading resistance in the electrolyte and shunt impedance of conducting traces become increasingly important. Spreading resistance (*R*_*s*_ ≈ ρ/2*d*) depends on electrode diameter *d* and medium conductivity ρ (Franks et al., 2005). Here, we estimate that our measurements with PBS (0.7 Ωm) include spreading resistance on the order of 100 kΩ. While this resistance will appear in impedance measurements, the presence of cells necessarily changes the situation. For example, proximal or engulfing cells will modify the spreading resistance. In this situation, it is more prudent to discuss the seal resistance, which can dramatically increase the magnitude of recorded extracellular potentials (Spira et al., 2018).

We estimate that our shunt capacitances were on the order of 100 pF, based on the area of the electrical trace (0.6–0.8 mm^2^), thickness of the silicon nitride insulator (400 nm) and its electric permittivity of 7 (Piccirillo and Gobbi, 1990), while the internal capacitance of our potentiostat adds another 10 pF. The shunt impedance is in parallel with the electrode and will shunt signals to ground (Robinson, 1968). The shunt capacitance produces a low pass filter, causing attenuation and distortion of higher frequency electrode signals both for recording and stimulation. For a more intuitive comparison, a 100 pF shunt has an impedance magnitude of 1.6 MΩ at 1 kHz.

Impedance spectra over the frequency range most relevant for extracellular electrophysiology (1–5000 Hz) had phase near −90°, showing predominantly capacitive behavior. The equivalent capacitances of these electrodes increased to ca. 400 pF with increasing amounts of PEDOT:PSS (Figure 2C). With an estimated surface area of ∼20 μm^2^, these capacitances are on the order of 2 mF/cm^2^, similar to those reported by Gerwig et al. (2012). Considering a thickness on the order of 100 nm, these results agree with volumetric capacitance of 100 F/cm^3^ calculated for PEDOT:PSS by density functional theory (Sahalianov et al., 2019).

### Optical and Scanning Electron Microscopy

**FIGURE 3:**
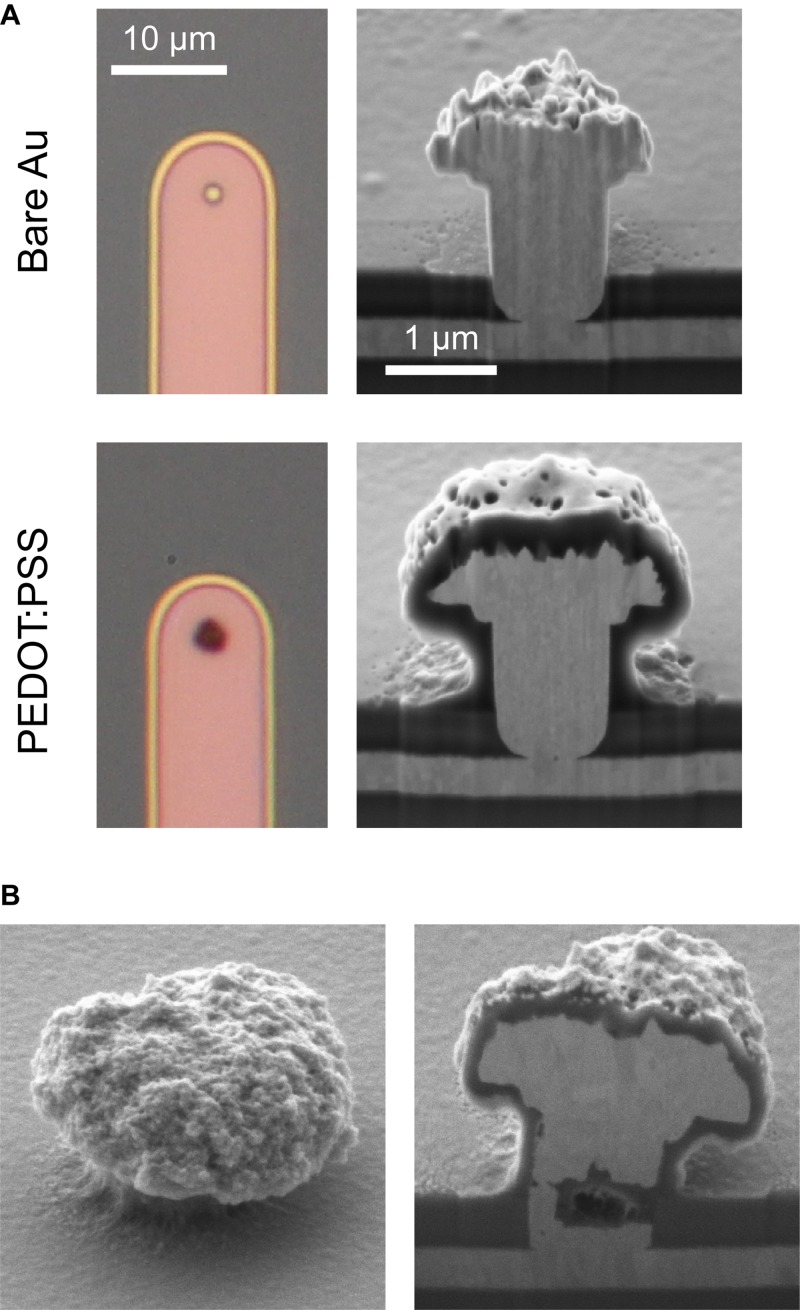
Optical and electron microscopy. **(A)** Optical micrographs (left) and SEM of FIB cross-sections of bare (above) and PEDOT:PSS-coated (below) gold ultramicroelectrodes. Electrodeposition of PEDOT:PSS produced a thin conformal layer. **(B)** Internal defects in an electrode were revealed by FIB cross-sections. Gold electroplating for this electrode used a current of −1.5 nA, in comparison to −0.4 nA for the electrodes in A. The scale bars in A apply to other optical and SEM images, respectively.

Optical microscopy indicated whether a ultramicroelectrode was bare gold or coated with PEDOT:PSS (Figure 3A), but could otherwise not reveal details. SEM revealed the dimensions and topography of the gold and PEDOT:PSS-coated electrodes (Figure 3). Our gold surfaces were rougher than other gold mushroom-shaped electrodes (Hai et al., 2009). This was caused in part by the 40°C temperature during electroplating; higher temperatures produced smoother surfaces but caused early delamination of our photoresist sacrificial layer. Cross-sections produced by FIB milling were necessary to reveal internal 3D structures, such as the thickness of the PEDOT:PSS layer or internal defects. The thickness of PEDOT:PSS was on the order of 100 nm.

### Stability

Optical microscopy showed no damage to the electrodes after 30 s of sonication. Impedance spectroscopy showed negligible differences in impedance after 14 h in ethanol or 30 s of air plasma, while submersion in PBS at 37°C increased impedance after two weeks (Figure 2D).

### Experimental Validation *in vitro*

**FIGURE 4:**
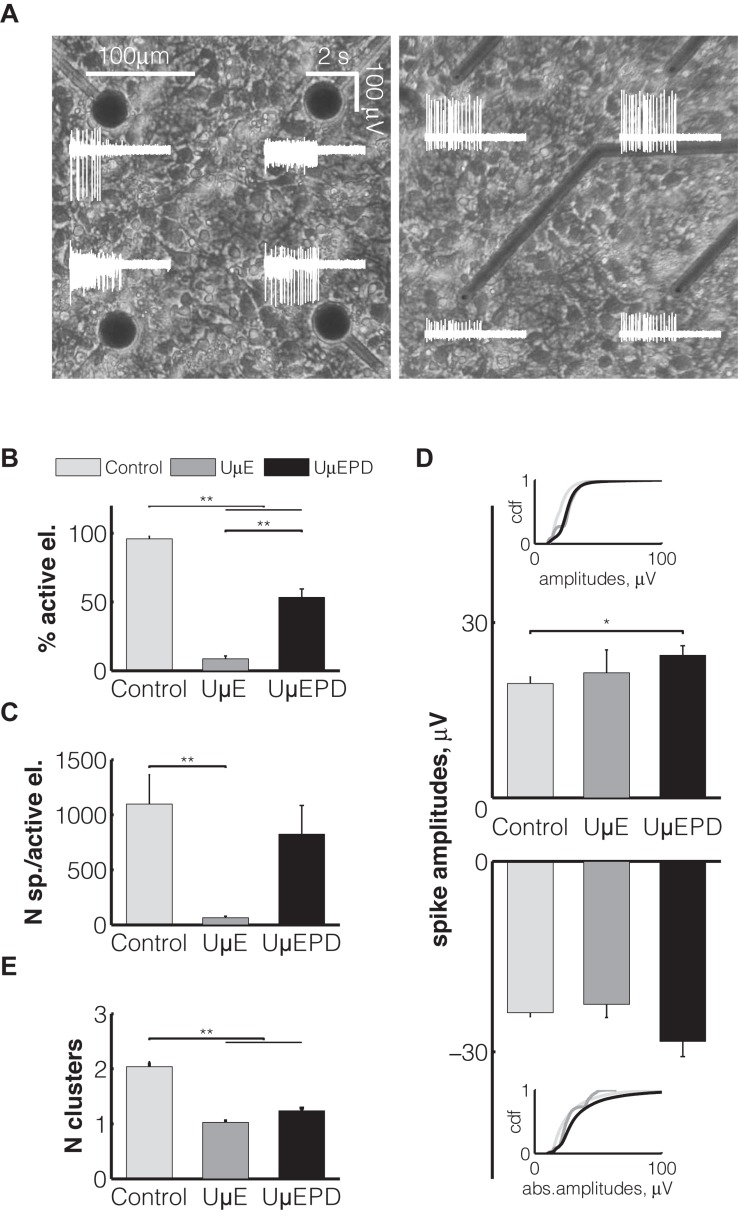
Elementary properties of different types of electrodes: conventional planar (light gray, *n* = 12 MEAs), gold ultramicroelectrodes (UμE, dark gray, *n* = 6 MEAs), PEDOT:PSS ultramicroelectrodes (UμEPD, black, *n* = 10 MEAs). Representative phase-contrast micrographs show cortical cells at the second week after plating on the glass surface with conventional planar electrodes (**A**, left) and with PEDOT:PSS ultramicroelectrodes (**A**, right). The white traces represent the electrical activity detected by the corresponding electrodes within an interval of 4 s. The percentage of active electrodes is represented as percentage of those electrodes detecting signals **(B)**. The average number of spikes per active electrode is displayed in **(C)**, together with the average value of detected amplitudes with positive (top) and negative (bottom) deflections **(D)**. The average number of different spike waveforms (clusters) detected by single electrode is displayed in panel **(E)**. Data are shown as the mean ± SEM and statistical significance between groups was assessed using a two-way ANOVA and *post-hoc* Fisher’s procedure.

The electrical impedance is an essential characteristic determining the sensitivity of microelectrodes employed for neuronal signal detection. For this reason, we began our investigation by testing the efficiency of neuronal signal recordings while taking into account that the impedance of ultramicroelectrodes is significantly higher than that of conventional larger planar electrodes. We tested 10 different rat neuronal cultures (see the section “Materials and Methods”) as we analyzed the recordings of spontaneous electrophysiological activity detected by ultramicroelectrodes (Figure 4A, right). As a control, we employed data obtained from the sister cultures, plated on commercial conventional MEAs (30 μm planar TiN; Figure 4A, left). As expected from *ex vivo* developing neuronal cell cultures, in all MEAs the electrical neuronal activity was characterized by sparse isolated spikes, starting from 7 days *in vitro* (DIV7). Spikes became spatially organized into episodic synchronous bursts, starting from DIV10 onwards (Figure 4A, white traces), suggesting a normal *ex vivo* synaptic and network development (Mahmud et al., 2014).

Our data revealed that less than 10% of (uncoated) gold ultramicroelectrodes were *active* (see the section “Materials and Methods”), detecting neuronal spontaneous signals (Figure 4B) during an observation interval of 30 min. We also observed that the number of spikes detected per *active* gold ultramicroelectrodes was 65 ± 13 (Figure 4C), significantly (*p* < 0.01) lower than the percentage of active electrodes (95 ± 2%) and number of spikes (1096 ± 268) detected by conventional planar microelectrodes, over the same time interval.

As soon as the coating with PEDOT:PSS was employed in our ultramicroelectrodes, it greatly improved the sensitivity of the ultramicroelectrodes, so that the fraction of active electrodes significantly (*p* < 0.01) increased and reached 53 ± 6% (Figure 4B), and so that the number of detected spikes by coated electrodes increased to 823 ± 261 (Figure 4C), making the differences with conventional electrodes non-significant, over the same time interval.

Regardless of the type of microelectrodes, spike waveforms with positive and negative peak deflections were detected. Their amplitudes varied over a wide rage, although in 50% cases they were below 35 μV. The largest amplitudes (i.e., up to ∼550 μV) were recorded with PEDOT:PSS ultramicroelectrodes. On average, positive (24 ± 2 μV) and negative (–28 ± 2 μV) spike peak amplitudes recorded with PEDOT:PSS ultramicroelectrodes were higher compared to those recorded by conventional planar electrodes (19 ± 1 μV and –24 ± 1 μV). Interestingly, for positive spike waveform amplitudes this difference was significant (*p* < 0.05) (Figure 4D). Moreover, the cumulative distribution function (Figure 4D, insets) of the absolute values of peak amplitudes detected by PEDOT:PSS ultramicroelectrodes was significantly shifted to the right, i.e., towards higher peak amplitudes, confirming the higher probability to detect higher spike amplitudes by PEDOT:PSS ultramicroelectrodes than by conventional MEAs.

During extracellular recordings by conventional techniques (MEAs), each electrode picks up signals from its spatial proximity and possibly from several neurons simultaneously. The diversity of spike peak amplitudes and waveform shapes requires spike sorting to identify the putative signal sources. Yet, the variability of intracellular action potentials (e.g., amplitude reduction of successive spikes during bursting) is also reflected in the extracellular signals produced by individual neurons, and this must be considered during spike sorting. In order to determine how many distinct neuronal units participated in spike generation during our extracellular recordings, we extracted specific features of the shape of the spikes, so that spike events with similar features were classified as belonging to the same group or “cluster”, following a state-of-the-art unsupervised spike sorting technique. After this procedure, each cluster became associated with a single putative isolated neuronal unit. Therefore, by using spike sorting and a well known clustering algorithm (see the section “Materials and Methods”), the spikes detected by planar MEAs, were routinely grouped on average into 2.0 ± 0.1 different clusters (i.e., neuronal units) per microelectrode, whereas those detected by gold ultramicroelectrodes were grouped in a significantly (*p* < 0.01) lower number of spike clusters (1.1 ± 0.0). This result was replicated also in the case of PEDOT:PSS ultramicroelectrodes (1.2 ± 0.1) (Figure 4E), suggesting that the smaller physical dimension of the ultramicroelectrodes determined an increase in the spatial selectivity of spike detection, for each electrode.

**FIGURE 5:**
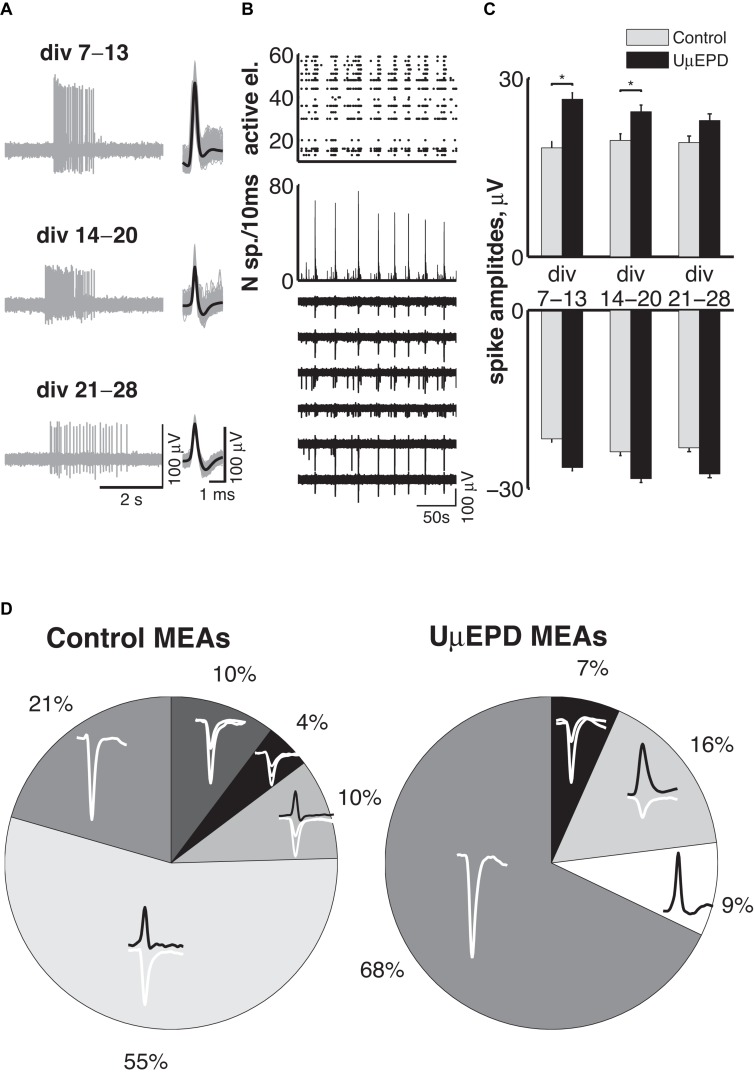
Biocompatibility, stability of ultramicroelectrodes, and spike-waveforms classification. Representative example of recordings from DIV7 up to DIV28 of spontaneous neuronal activity with PEDOT:PSS ultramicroelectrodes (**A**, left) and extracted spike waveforms (**A**, right); periodic synchronized network behavior within 200 s is presented by spike-time (black dots) raster plot over all PEDOT:PSS ultramicroelectrodes (**B**, top), histogram of spike time computed for 10 ms intervals across all microelectrodes (**B**, middle) and extracellular recording with six PEDOT:PSS ultramicroelectrodes (**B**, bottom) during corresponding time; the distribution of the average amplitudes with positive (**C**, top) and negative (**C**, bottom) deflections over 3 weeks of neuronal network development detected with control (gray bars, *n* = 12 MEAs) and PEDOT:PSS ultramicroelectrodes (black bars, *n* = 10 MEAs) microelectrodes, data are shown as mean ± SEM; classification of spike waveforms detected by planar microelectrodes (**D**, left, *n* = 4 MEAs) revealed that the majority (79%) of planar electrodes detects signal with several different spike shapes simultaneously, whereas the majority of PEDOT:PSS ultramicroelectrodes (77%) microelectrodes registered only single waveform of spikes (**D**, right, *n* = 7 MEAs).

One more necessary requirement for microelectrodes is the long-time stability of recordings. We tested our PEDOT:PSS ultramicroelectrodes and evaluated their non-invasive properties vs. planar control microelectrodes, by performing long-term recordings (i.e., over successive days) during the development of network activity *in vitro*. As for planar electrodes of conventional MEAs, PEDOT:PSS ultramicroelectrodes detected both isolated asynchronous spikes as well as episodic synchronized burst of spikes, over 3 weeks of culturing *in vitro* (Figure 5A, left panel). During this time, the shape of detected spikes remained stable (Figure 5A, right panel). Synchronized activity across whole neural network was detected for all tested PEDOT:PSS ultramicroelectrode MEAs (*n* = 10) and a typical raster plot (Figure 5B top), integrating network firing rates (Figure 5B middle) and raw voltage traces (Figure 5B bottom), revealed the dynamics of functional networks *in vitro*, similar to conventional planar electrodes. Long-term monitoring of neuronal network activity throughout the second, the third and the fourth weeks *in vitro* revealed that both positive (Figure 5C top, conventional planar: 18 ± 1 μV; 19 ± 1 μV; 19 ± 1 μV; PEDOT:PSS ultramicroelectrodes 27 ± 4 μV; 24 ± 2 μV; 23 ± 2 μV) and negative (Figure 5C bottom, conventional planar: –22 ± 1 μV; –24 ± 1 μV; –23 ± 1 μV; PEDOT:PSS ultramicroelectrodes: –26 ± 3 μV; –28 ± 2 μV; –28 ± 2 μV) peak amplitudes did not change. Despite the amplitudes recorded with PEDOT:PSS ultramicroelectrodes being slightly higher than the ones detected with planar electrodes, a significant difference (p < 0.05) was observed only for positive peak amplitude spikes, during the second and third weeks *in vitro*.

The majority of spike waveforms detected with PEDOT:PSS ultramicroelectrodes had single negative (68%) or single positive (9%) peak deflections (Figure 5D, right), whereas only 21% of conventional planar electrode detected single spikes waveforms with negative peak deflection (Figure 5D, left). The majority of planar electrodes instead detected spikes with biphasic (positive and negative) deflections (65%) simultaneously, with single positive peak spikes never detected by planar electrodes.

## Discussion

In this work, we aimed to demonstrate the possibility of novel MEA fabrication with low impedance ultramicroelectrodes, exhibiting in comparison to standard MEAs an adequate signal-to-noise ratio, an improved selectivity for single units and a proper stability throughout long-term recordings.

The majority of previous studies in the literature suggest that electrode impedance has an important impact on signal-to-noise ratio (Ludwig et al., 2011; Scott et al., 2012; Chung et al., 2015) and therefore on sensitivity to detect spikes, although the opposite point of view has been expressed (Neto et al., 2018). Our data demonstrate that the electrode impedance is crucial for spike detection in extracellular recordings and that a simple and reproducible technique, such as PEDOT:PSS coating, leads to a stable, biocompatible, and significantly increased effectiveness of neural signal recording.

Notably, in the absence of the coating, gold ultramicroelectrodes per se have an expected impedance magnitudes above 10 MΩ at 1 kHz, as estimated by gold’s double-layer capacitance of ∼0.7 pF/μm^2^ (Piela and Wrona, 1995). In this circumstance, the lower shunt impedance of the insulator prevented an independent measurement of the impedance of gold ultramicroelectrodes. More importantly, recording or stimulation with such uncoated ultramicroelectrodes is severely attenuated due to substantial shunting of the signals to the grounded bath.

Nonetheless, surprisingly large signals have been previously reported with similar (uncoated) gold ultramicroelectrodes (Spira and Hai, 2013; Shmoel et al., 2016). We speculate that these early results might be the consequence of a minimization of this effect with optimized dimensions of electrical traces and of the insulator permittivity. We estimate that, in our case, the shunt capacitance could have been reduced by a factor of 16 upon narrowing our electrical traces to 5 μm, which would still not compromise the fabrication process.

The challenge of shunt capacitance for gold ultramicroelectrodes emphasizes the benefit of low impedance PEDOT:PSS coating. Packaging (e.g. gluing of culture chambers on *in vitro* MEAs) can also increase the impedance of microelectrodes (McDonald et al., 2017). Since PEDOT:PSS deposition is performed later in the fabrication process and includes electrochemical cleaning, this problem is entirely avoided.

In our biological experiments, we have overall demonstrated that the number of *active* (or recording) PEDOT:PSS ultramicroelectrodes is smaller than in conventional MEAs but still adequate for capturing the synchronized network activity emerging spontaneously in neuronal networks developing *ex vivo* (Figures 5A,B). The 200 μm spacing between electrodes allowed us to keep cells naturally distributed in culture without the need for enforcing a patterning on their substrate adhesion, and made cell guidance not pivotal in contrast to previous contributions to the literature (Santoro et al., 2013). For this reason, we feel we might have avoided the alteration in the neurites growth pattern (Hai et al., 2009) and, as a result, obtained a more naturalistic *in vitro* network connectivity.

This is also supported by the average number of spikes, detected with single PEDOT:PSS ultramicroelectrodes, as it was tendentially lower than with conventional MEA electrodes. A reduction could be expected due to the electrode selectivity, suggested by our results: as PEDOT:PSS ultramicroelectrodes often detected signals from a single neuronal unit, in contrast to the typical multiunit recordings of conventional MEA electrodes. Therefore, our results seem to favor PEDOT:PSS ultramicroelectrodes as a high-selectivity mean of recording neural signals, with reduced needs for spike sorting. Of course, only future tests *in vivo* will conclusively establish whether a clear advantage for a less ambiguous spike sorting might be achieved with the ultramicroelectrodes. If confirmed, recorded data of such new experiments might require lower sampling frequencies, as an analysis of the spike shapes would not be strictly required, leading as a side effect to a much lower volume of data produced for each experimental session.

Signal amplitudes detected with PEDOT:PSS ultramicroelectrodes were comparable with those detected with conventional electrodes, meanwhile, a recent experiment (Shmoel et al., 2016) showed that gold ultramicroelectrodes make possible the recording of signals with amplitudes reaching several mV (approx. 2% of reported neurons had amplitudes >1 mV, with only 2/695 neurons above 2 mV). These positive results suggest a promising potential for these kind of 3D electrodes, indicating the directions for further improvement of technology, e.g., the neuron–microelectrode junction.

It has been already shown (Fendyur et al., 2011) that rat hippocampal cells are able to engulf micro protruding electrodes with size about 1–2 μm, creating tight contacts, required for successful signal detection. However, such a systematic study did not cover the important aspect of how long and how stable is a similar detection over time. Then, by our results we offer additional evidences, suggesting that PEDOT:PSS ultramicroelectrodes are capable of recording under stable conditions the typical synchronized activity, developing *ex vivo* in neuronal networks over 4 weeks *in vitro*. Indeed, the averaged spike amplitudes were highly stable throughout the recording sessions. Even considering the capability to measure intracellular-like action potentials with protruding nanowires (Robinson et al., 2012; Xie et al., 2012; Dipalo et al., 2017; Abbott et al., 2019; Desbiolles et al., 2019), a key feature and the main advantage of microelectrode arrays (i.e. long-term noninvasive recordings) is not achieved by the nanowires. Also with a quantitative analysis of the number of penetrating nanowires, it had been shown (Aalipour et al., 2014) that the lipid bilayer and the actin cytoskeleton act as barriers to accessing the cell cytosol, so that a (chemical) poration might be still insufficient to increase long-term access to cells.

We emphasize that additional knowledge is required to achieve reliable intracellular-like recordings in mammalian cell types and, ultimately, *in vivo* (Spira et al., 2018). Intimate contact and surface recognition are necessary for achieving an effective high seal resistance and a low junctional membrane resistance. We recommend correlation of local surface chemistry (e.g., by X-ray photoelectron spectroscopy) with ultrastructural and molecular biological behavior (e.g., by immunochemical staining and FIB tomography). It will be in fact very important to distinguish between the macroscopic and microscopic properties, such as the different interaction with PEDOT:PSS of molecules (e.g., PEI) used for increasing cell adherence to the larger insulating area (e.g., silicon nitride).

## Conclusion

In this paper, we presented and experimentally validated a novel type of MEAs that, to our knowledge, represent currently the best-reported combination of small electrode size and low electrical impedance, under the overall perspectives of highly parallel, non-invasive, intracellular-like recordings by extracellular MEAs. The fabrication we described is simpler than what is reported in the literature, relying only on contact lithography and avoiding electron beam lithography or advanced nanofabrication of nanowire electrodes.

The present study finally demonstrates that the presence of PEDOT:PSS ultramicroelectrodes did not alter neuronal network biological properties *in vitro*. The coating by a conducting polymer significantly improved the quality of extracellular recordings, when compared to uncoated ultramicroelectrodes. We conclude that ultramicroelectrodes offer further perspectives in improving the neuron–electrode interface, while enhancing the quality and sensitivity of MEAs recordings and thus representing a promising tool for our study of neuronal networks.

## Data Availability Statement

The datasets generated for this study are available on request to the corresponding authors.

## Ethics Statement

The animal study was reviewed and approved by Animal Ethics Committee of the University of Antwerp.

## Author Contributions

Conceived and designed the research: RS and MG Performed the experiments, fabricated the devices: AM, PJ, CB, KG, GH, BS, and RS. Wrote the manuscript: AM, PJ, RS, and MG.

## Conflict of Interest

RS is employed by NMI TT GmbH, which sells microelectrode arrays. The remaining authors declare no competing financial interests. CB and KG contributed to this work while affiliated with NMI at the University of Tübingen, and are currently employed by joimax GmbH and Bosch Sensortec GmbH, respectively.
